# Separating the present and the future

**DOI:** 10.7554/eLife.43339

**Published:** 2018-12-04

**Authors:** Qing Yu, Bradley R Postle

**Affiliations:** 1 Department of PsychiatryUniversity of Wisconsin-MadisonMadisonUnited States; 2 Department of PsychologyUniversity of Wisconsin-MadisonMadisonUnited States

**Keywords:** working memory, visual attention, cognitive control, multivariate pattern decoding, category representations, prospective memory, Human

## Abstract

The brain stores information that is needed immediately and information that will be needed in the future in different ways.

**Related research article** van Loon AM, Olmos-Solis K, Fahrenfort JJ, Olivers CNL. 2018. Current and future goals are represented in opposite patterns in object-selective cortex. *eLife* **7**:e38677. doi: 10.7554/eLife.38677

Imagine you are in a grocery store without a shopping list. Even though you need to purchase multiple items, you still need to select them one by one. How does the brain distinguish between the most relevant information from everything else on our mind? In particular, is information about the item you intend to buy first stored in your 'working memory' in the same way as information about the other items?

A common method used to study memory processing in the human brain is functional magnetic resonance imaging (fMRI). Moreover, researchers often use machine learning techniques, such as multivariate pattern analysis, to decode information from the fMRI data. Typically, if multivariate pattern analysis is able to discriminate between different pieces of information based on their activity patterns in the brain, this is interpreted as evidence for an 'active' representation of the information. Alternatively, a failure to decode might suggest there is no active representation of it.

It is generally acknowledged that information that is immediately relevant is actively represented in working memory, but it remains unclear if this is also true for information that will be needed in the future (that is, for prospectively relevant information: see, for example, LaRocque et al., 2013; Lewis-Peacock et al., 2012). One possibility is that the latter is stored in an ‘activity-silent’ manner, due to a transient change in the strength of the synaptic connections between neurons (see, for example, Barak and Tsodyks, 2014). Such information can usually not be detected by traditional fMRI measurements, unless the network is stimulated to reactivate the ‘activity-silent’ information (Rose et al., 2016; Wolff et al., 2017). Alternatively, prospectively relevant information may be transferred to brain regions that are different from those where immediately relevant information is held (see, for example, Christophel et al., 2018).

Now, in eLife, Christian Olivers and colleagues of the Vrije Universiteit Amsterdam and the University of Amsterdam – Anouk van Loon, Katya Olmos Solis and Johannes Fahrenfort – report evidence for a third possibility, namely that prospectively relevant information is represented actively, but in a recoded format (van Loon et al., 2018).

To explore how working memory distinguishes between immediately relevant and prospectively relevant information, volunteers were asked to perform two visual search tasks. In the first experiment, they consecutively viewed a flower and another object (either a cow, a dresser or a skate), and the order was manipulated between trials. A cue during the initial presentation indicated which image would be relevant for the first (imminent) or the second (prospective) search. Then, depending on the trial (current vs. prospective), they had to first search for the target flower in an array of flowers and then search for the other target (e.g., a cow in an array of different cows), or vice versa. During the tasks, the researchers used fMRI to measure a region of the brain involved in categorizing objects, called the posterior fusiform cortex.

The researchers found that before the volunteers knew which of the two images would be the first search target, both were actively represented in a similar way in working memory. However, once one of the images was designated as immediately relevant, the representations diverged. Although both were still actively represented, the patterns of the two stimuli were the inverse of each other, as indicated by multivariate pattern analysis and another technique called representational dissimilarity analysis.

Moreover, after the first search, when the prospectively relevant information became immediately relevant, its representation in the brain 'flipped' back to its original pattern. In a second experiment, the researchers found that this reversed pattern only happened if the information was prospectively relevant; if the volunteer was told that the information was no longer relevant, it was lost from working memory.

As van Loon et al. acknowledge, other research groups have made similar discoveries using different kinds of tasks and different kinds of stimuli. Together all these results have important implications for our understanding of the representation of information in working memory. Therefore, a key goal for future research will be to clarify the circumstances under which prospectively relevant information is re-represented – relative to immediately relevant information – in a different pattern, in a different region of the brain, or a different state.
